# Retraction: Overexpressed EDIL3 predicts poor prognosis and promotes anchorage-independent tumor growth in human pancreatic cancer

**DOI:** 10.18632/oncotarget.28909

**Published:** 2026-09-23

**Authors:** Shu-Heng Jiang, Yang Wang, Jian-Yu Yang, Jun Li, Ming-Xuan Feng, Ya-Hui Wang, Xiao-Mei Yang, Ping He, Guang-Ang Tian, Xiao-Xin Zhang, Qing Li, Xiao-Yan Cao, Yan-Miao Huo, Min-Wei Yang, Xue-Liang Fu, Jiao Li, De-Jun Liu, Miao Dai, Shan-Yun Wen, Jian-Ren Gu, Jie Hong, Rong Hua, Zhi-Gang Zhang, Yong-Wei Sun

**Affiliations:** ^1^Shanghai Medical College of Fudan University, Shanghai, P.R. China; ^2^State Key Laboratory of Oncogenes and Related Genes, Shanghai Cancer Institute, Ren Ji Hospital, School of Medicine, Shanghai Jiao Tong University, Shanghai, P.R. China; ^3^Department of Biliary-Pancreatic Surgery, Ren Ji Hospital, School of Medicine, Shanghai Jiao Tong University, Shanghai, P.R. China; ^4^Department of Urology, Shanghai No.5 People’s Hospital, Fudan University, Shanghai, P.R. China; ^5^Department of Liver Surgery, Ren Ji Hospital, School of Medicine, Shanghai Jiao Tong University, Shanghai, P.R. China; ^6^Division of Gastroenterology and Hepatology, Ren Ji Hospital, Shanghai Institution of Digestive Disease, Key Laboratory of Gastroenterology and Hepatology, Ministry of Health, State Key Laboratory of Oncogene and Related Genes, Shanghai Jiao-Tong University School of Medicine, Shanghai, P.R. China; ^7^Department of Obstetrics and Gynecology, Shanghai Jiao Tong University Affiliated Sixth People’s Hospital, Shanghai, P.R. China; ^*^These authors contributed equally to this work

**This article has been retracted:** Oncotarget has concluded its investigation of this paper, and the following issues were discovered:

- Figure 1E, presenting photographs of the EDIL3 immunoreactivity in normal pancreas (NP), chronic pancreatitis (CP) and PDAC tissues in TMA1, includes identical images for NP Case 1 and NP Case 2. Additionally, the TMA1/PDAC image for Case 2 overlaps with Figure 1A of an unrelated, recently retracted 2015 article [1].
- Figure 3E: The FACS plots for SW1990 Sh-1 and Sh-2 clones are duplicates, as are the BxPC-3 panels.
- Figure 4B: The Tube formation assay image of BxPC-3CM overlaps with the image in Figure 6B of article [2], which shares several authors with this paper.
- Figure 4E: The Colony formation assay of PANC1 cells contains duplicated images for Control and 10nM treatment groups. Additionally there is an overlap between SW1990/sh-2 image and Figure 3G AsPC1-Ctrl image.
- Supplementary Figure 1D: SW1990 cells invasion assay Sh-Ctrl image overlaps with sh-1 image.
- Supplementary Figure 1E: BxPC-3 cells starvation-induced apoptosis assay flow cytometry plots of Sh-Ctrl and sh-1 are duplicates.

While the authors initially acknowledged the internal duplications and stated they did not impact the experimental conclusions, there has been no response to our subsequent requests for the provision of original data. Given the extensive number of duplications, the editors can no longer be confident that the overall conclusions remain unaffected. Consequently, the editorial decision has been made to retract the paper and the authors have been informed.

The first author Dr. Shu-Heng Jiang stated: “We acknowledge that these errors, though traceable to our original data, which we are prepared to provide upon request, undermine confidence in the article. Given the extent of the issues, we have decided to voluntarily retract this paper. We offer our sincerest apologies for any inconvenience or harm caused.”

Original article: Oncotarget. 2016; 7:4226–4240. 4226-4240. https://doi.org/10.18632/oncotarget.6772
